# Optimism’s Explicative Role for Chronic Diseases

**DOI:** 10.3389/fpsyg.2016.00295

**Published:** 2016-03-02

**Authors:** Giulia Avvenuti, Ilaria Baiardini, Anna Giardini

**Affiliations:** ^1^Psychology Unit, Scientific Institute of Montescano, Salvatore Maugeri Foundation, Istituto di Ricovero e Cura a Carattere ScientificoMontescano, Italy; ^2^Allergy and Respiratory Diseases Clinic, Department of Internal Medicine, University of Genoa – IRCCS AOU San Martino-ISTGenova, Italy

**Keywords:** optimism, health status, protective behaviors, chronic diseases, self-management

## Abstract

The increasing interest about dispositional optimism’s role in health status and its positive modulating effect on health outcomes has led to a remarkable scientific production in the last decade. To date lot is known for which diseases optimism is relevant, instead much less is known about how optimism interacts with other factors, both biological and psychological, in determining health status. The aim of this mini review is to explore the literature derived from clinical and experimental research assessing the associations between dispositional optimism and health status. Dispositional optimism can be considered as facet of personality that is cognitive in nature which holds the global expectation that the future will be plenty of good events. Optimists view desired goals as obtainable, so they often confront adversities in active manners resulting in perseverance and increased goal attainment. Only studies that explicitly included optimism and health outcomes, as measurable variables, and that reported a clear association between them have been reviewed. Cancer, cardiovascular disease, respiratory failure, and aging with multimorbidity were considered. Among the possible explicative hypotheses, two seem to best describe results: optimism may have a direct effect on the neuroendocrine system and on immune responses, and it may have an indirect effect on health outcomes by promoting protective health behaviors, adaptive coping strategies and enhancing positive mood. The research on optimism and health status has already shed light on important mechanisms regarding chronic diseases’ management, however, further studies are needed to deepen the knowledge.

## Introduction

In folk wisdom optimists are those who expect good things to happen to them, whereas pessimists are those who expect bad things to happen to them. Folk psychology has long affirmed that people can be differentiated in terms of optimism or pessimism (Carver et al., 2010).

Although researches generally agree upon the overall definition of optimism, optimism has been conceptualized in different ways. Tiger described optimism as a mood or a mental status associated with the expectation of a desired event in the future since it provides advantages or pleasures (Tiger, 1979). In this perspective optimism is a part of human nature: it is the psychological mechanism that drives human evolution entailing thinking about the future. From another point of view, Scheier and Carver (1985) defined optimism as a general tendency to expect that one will experience positive versus negative events in the future, similar to a personality trait. Optimism was then theorized as a stable organization of affects and cognitions that can shape the relation between people and reality, determining one’s self-perception, expectations, and sense of agency.

### Different Conceptualizations of Optimism

In the growing literature on optimism, some differences can be found between *unrealistic* and *realistic optimism, attributional*, and *dispositional optimism* (Anolli, 2005). *Unrealistic optimism* consists of a set of cognitive mechanisms (i.e., inappropriate beliefs) that make one think, as an example, to be immune from health’s threats. College’s students, for example, when asked to estimate their own likelihood, in comparison with peers, of experiencing divorce or substance abuse problems in adulthood, underestimate their risk. Similarly, students compared to peers overestimate their likelihood of living until 80 years old (Weinstein, 1980, 1989). Shepperd et al. (2013), aware of contradictory unrealistic optimism’s definitions, distinguished between two types of unrealistic optimism: the absolute one and the comparative one, both expressing at the individual and group level. Moreover they provided interesting cues on psychological functioning describing the role of unrealistic optimism in the process of underestimation of personal risk for some events and of overestimation of personal risk for others (Shepperd et al., 2013). This phenomenon is a kind of overconfidence, a wishful thinking, an illusion that alters the perceived vulnerability, an error in judgment, similar to children’s magic thought. According to Sharot, unrealistic optimism is a general human tendency that occurs when people have to update information: if the new information disconfirms one’s expectation it will not be integrated (Sharot et al., 2011). This valence-dependent asymmetry seems to have an adaptive function, maintaining well-being, since it enhances explorative behaviors and reduces stress and anxiety (Sharot, 2011; Sharot and Garrett, 2016). Indeed, mild depression was related to realism, whereas depression was related to the absence of optimistic bias in information updating, and this absence was correlated to symptoms severity (Strunk et al., 2006; Korn et al., 2014). Finally, unrealistic optimism seems to be influenced by culture of belonging: being part of an individualistic or a collectivistic culture, or belonging to a culture where the economic gap is wide, may determine differences in the ways in which individuals judge the likelihood of an event. However, data are still controversial, with different authors debating the presence/absence of unrealistic optimism in different cultures (Joshi and Carter, 2013). On the other side, *realistic optimism* involves anticipating good things to happen in the future, but taking in the meantime into account contextual information; therefore a balance between expectancy, goal’s value and effort is maintained (Scheier and Carver, 1992; Carver and Scheier, 1998; Higgins, 2006). For that reason, realistic optimism seems to be essential for mental and physical health (Davis and Asliturk, 2011).

**FIGURE 1:**
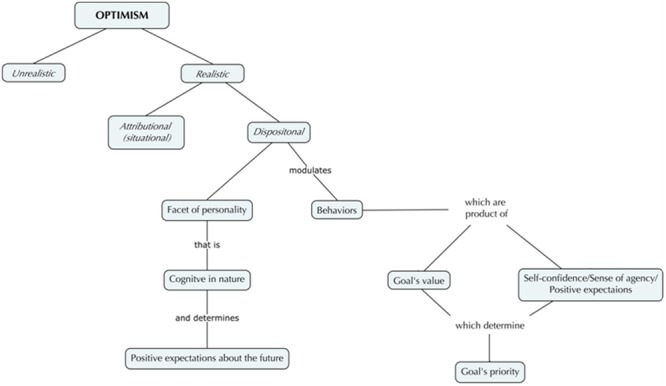
**Conceptual map on optimism’s categorization**.

In the framework of realistic optimism two other different interpretative models can be found: *attributional (situational)* and *dispositional optimism*. The former (*attributional optimism* theory) originated from a reformulation of the learned helplessness model: its explanatory style was described as an individual characteristic, based on past events’ interpretations, that may account for inter-individual differences in responding to events (Abramson et al., 1978). Subsequently, optimism was formulated as belonging to the explanatory style: those who explain bad events as having unstable, specific, and external causes are described as optimists; those who explain the same negative events as having stable, global, and internal causes are described as pessimists. Moreover, optimists tend to explain positive events as global, stable, and internal, while pessimists explain the same good events as unstable, specific, and external (Buchanan and Seligman, 1995; Peterson, 2000). The latter refers to Scheier and Carver (1992) studies on *dispositional optimism*, considered as a personality trait which holds the global expectation that the future will be plenty of good events (**Figure 1**). To be more precise, dispositional optimism is a facet of personality that is cognitive in nature, that contains expectancies about the future and that is linked to expectancy-value models of motivation (Carver et al., 2010; Carver and Scheier, 2014). Studying optimism, according to these authors, is to display how personal goals are turned into behaviors. The model assumes that life mainly concerns pursuing goals and that behaviors are the product of values combined with expectancies (Rasmussen et al., 2006). The first component of this model is the value that a goal acquires: the more important is a goal to a person, the more commitment and effort are employed in reaching the desired outcome. Secondly, expectancies about the future are determined by the confidence that the goal can be attained: if one is confident about her/his success in attaining goals, effort continues; if one is not, effort is disengaged. Optimists view desired goals as obtainable, so they confront often adversities in active manners resulting in perseverance and increased goal attainment. Furthermore, dispositional optimism influences how people engage their efforts in pursuing goals on the basis of goals’ priority, thus determining individual well-being (Scheier and Carver, 2003; Wrosch et al., 2003a, 2007): for high-priority goals optimists invest resources maximizing the probability of attainment, whereas for low-priority goals they disengage and do not increase goal-oriented activities (Wrosch et al., 2003b; Geers et al., 2009, 2010). In a general way, optimists appear to be approach copers (both problem-focused and emotion-focused), whereas pessimists appear to be avoidant copers. Furthermore, optimists disengage more easily than pessimists from an unattainable goal and reengage in a new one, that assume priority, and relocate resources (Nes and Segerstrom, 2006; Carver et al., 2010). Success’ expectation, typical of optimists, is likely to generate positive affects (Rasmussen et al., 2006). Indeed, among the multiple factors interaction, dispositional optimism seems to be the most influential in predicting psychological well-being, protecting individuals from anxiety and depression when giving up from unattainable goals is the best thing to do (Wrosch and Sabiston, 2013; Lam et al., 2016).

Dispositional optimism could be then considered a marker of resilience: it is related to positive mood, to perseverance and effective problem solving, to personal success, to good health status and to long life. In contrast, pessimism is related to depression, failure, social estrangement, morbidity, and mortality (Scheier and Carver, 1985; Peterson, 2000; Rasmussen et al., 2009).

Taking into account its influence on the way people perceive and conduct their lives (Carver et al., 2010), dispositional optimism has been related to health and pathological processes.

The aim of this mini review is to explore the literature derived from clinical and experimental research assessing the associations between optimism and health status. Studies investigating dispositional optimism’s role in adjustment to chronic diseases and health outcomes are now remarkable and will be described in the following paragraphs (**Table 1**).

**Table 1:** Articles reviewed dealing with dispositional optimism.

Article	Study design	Sample size	Health condition considered	Optimism assessment tools	Psychological conditions considered other than optimism and their assessment tools
**Optimism and cancer**					
Rajandram et al., 2011	Observational cross-sectional	50 tumor free pts	Oral cavity cancer	LOT-R	Anxiety – HADS Depression – HADS Trait hope – AHS
Gustavsson-Lilius et al., 2012	Observational longitudinal	147 couples (pt and caregiver)	Cancer	LOT-R	Anxiety – EMAS-State Depression – BDI-14 Sense of coherence – SOC-12
Lam et al., 2016	Observational longitudinal	172 female pts	Breast cancer	LOT-R	Psychological distress – HADS Positive affect – PANAS Goal adjustment – Goal Adjustment Scale Dispositional hope – Chinese Hope Scale
Orom et al., 2015	Observational cross-sectional	1425 male pts	Prostate cancer	LOT-R	Decision-making, self-efficacy – Tailored 3-item questionnaire Confidence in cancer control – “I am able to feel like a man” Masculine identity – BRS Emotional distress – Distress Thermometer (VAS)
O’Brien and Moorey, 2010	Systematic review		Cancer		
Shand et al., 2015	Systematic review		Cancer		
**Optimism and cardiovascular diseases**					
Scheier et al., 1999	Prospective inception cohort design	309 pts (216 male, 93 female)	Coronary artery bypass graft surgery (CABG)	LOT-R	Self-esteem – Rosenberg Self-esteem Scale Depression – CES-D Neuroticism – EPQ
Myaskovsky et al., 2006	Observational longitudinal	199 pts (121 male, 78 female)	Heart or lung transplant	LOT	Health Related Quality of Life – SF-36 Caregiver/friend support – 12 tailored items Religiosity – Three tailored items Coping strategies – Brief-COPE
Nabi et al., 2008	Observational longitudinal cohort	1,021 pts (212 male, 809 female)	Hypertension	LOT-R	Sense of coherence – SOC-13 Hostility – Finnish Twin Study Scale of Hostility Anxiety – Anxiety-Trait Scale
Tindle et al., 2009	Observational	97,253 women	All causes mortality	LOT-R	Cynical hostility – Cook-Medley Questionnaire
Boehm et al., 2011	Observational	7,942 adults (5,488 male, 2,454 female)	Incident Coronary Heart Disease	“Over the next 5–10 years, I expect to have many more positive than negative experiences,”	Emotional vitality – Tailored five items Psychological ill-being – SF-12
Kim ES et al., 2011	Observational	6,044 adults (2,542 male, 3,502 female)	Stroke risk	LOT-R	Self-rated health status – SF-36
Tindle et al., 2012	Observational prospective cross-sectional	430 pts (260 male, 170 female)	Post CABG	LOT-R	HRQoL – SF-36 Depression – PHQ/HRS-D Anxiety – Primary Care Evaluation of Mental Disorders Social support – Perceived Social Support Scale Adherence – Ziegelstein Healthy Lifestyle Questionnaire
Kim et al., 2014	Observational	6,808 adults (2,792 male, 4,016 female)	Incident CHD	LOT-R	Depression – CES-D Anxiety – BAI
Mahler and Kulik, 2010	Observational prospective longitudinal	212 male pts	Recovery from CABG	LOT-R	
Ronaldson et al., 2015	Observational prospective	369 pts(296 male, 73 female)	Acute coronary syndrome (ACS)	LOT-R	Depression – BDI Health status – SF-12
Chida and Steptoe, 2008	Systematic review		Cardiovascular disease		
Tindle et al., 2010	Review		Cardiovascular disease		
DuBois et al., 2012	Review		Cardiovascular disease		
Boehm and Kubzansky, 2012	Review		Cardiovascular disease		
DuBois et al., 2015	Systematic review		Cardiovascular disease		
**Optimism and respiratory failure**					
Alberto and Joyner, 2008	Observational	68 pts (26 male, 37 female)	Chronic obstructive pulmonary disease (COPD)	LOT-R	Hope – Herth Hope Index Self care – Alberto COPD Self-Care Behavior Inventory
Popa-Velea and Purcarea, 2014	Observational	54 pts (28 male, 26 female)	COPD	LOT-R	Self-efficacy – COPD Self-Efficacy Scale Well-being – Quality of WellBeing Scale
**Optimism and multiple chronic conditions**					
Kostka and Jachimowicz, 2010	Observational cross-sectional	324 elderly (73 male, 251 female)	healthy community-dwelling elderly, independent elders who voluntarily decided to live in veteran home and inhabitants of a long-term care home	LOT-R	health locus of control – MHLC self-efficacy – generalised self-efficacy scale (GSES) Quality of Life – Euroqol 5D questionnaire, the Nottingham health profile (NHP) and the satisfaction with life scale (SWLS)
Pitkala et al., 2004	Observational cross-sectional longitudinal	491 old–old subjects (137 male, 354 female)	General aged population	Tailored five item questionnaire	Cognitive impairment – Clinical Dementia Rating Scale and Mini Mental State Examination Depression – Zung depression scale Major depression – Diagnostic and Statistical Manual III
Giltay et al., 2007	Observational longitudinal	887 elderly community-living men	General population	Tailored four item questionnaire	
Luger et al., 2009	Observational cross-sectional, longitudinal	160 old adults	Osteoarthiritis	LOT-R	Social support – 19-item Medical Outcomes Study – Social Support Survey
					Social strain – Test of Negative Social Exchange (TENSE) Life satisfaction – Life Satisfaction Inventory (LSI)
Rius-Ottenheim et al., 2012	Observational longitudinal	416 old men	General population	Tailored four item questionnaire	Loneliness – 11-item loneliness scale of De Jong Gierveld
Gison et al., 2014	Observational cross-sectional	70 pts (47 male, 23 female) 70 healthy subjects (40 male, 30 female)	Parkinson’s disease	LOT-R	Quality of Life – WHO-5 Well-being Index (WHO-5) Anxiety and depression – HADS

### Dispositional Optimism and Health

#### Optimism and Cancer

Optimism seems to be strongly associated to individual responses to cancer: it fosters emotional and behavioral adjustment, it is linked to low levels of anxiety and depression symptoms both at an individual and relational level.

In O’Brien’s systematic review positive attitude (optimism and active coping) resulted associated with better emotional adjustment in later stages of the disease (O’Brien and Moorey, 2010), even if data are still controversial and few evidence based studies are available. Still, a recent meta-analysis showed that post-traumatic growth, intended as positive psychological changes occurring after a trauma, is related with optimism and positive coping strategies (i.e., positive reappraisal, religious coping, seeking social support; Shand et al., 2015).

Baseline dispositional optimism, assessed at the time of diagnosis, in cancer patients predicted less depressive and anxious symptoms at 8 months follow-up, and an association between higher partner optimism at baseline and lower patient anxiety at follow-up was found (Gustavsson-Lilius et al., 2012). Rajandram found that oral cavity cancer outpatients that had higher levels of hope and optimism at baseline, reported lower levels of anxiety, and depression at follow-up controls. A possible explanation is that both the hope regarding positive expectations about future actions engagement and trait optimism regarding positive expectations about environmental circumstances, may have led to a more efficient engagement in adaptive coping strategies, which in turn leads to a better psychological health (Rajandram et al., 2011). Moreover, consistent with previous studies (Nekolaichuk and Bruera, 2004; Thornton and Perez, 2006; Utne et al., 2008; Rajandram et al., 2011), high optimism and high disengagement from unattainable goals in women with advanced breast cancer diagnosis were associated with low anxiety and low depression, whereas high levels of hope were associated with new and alternative goals reengagement at 12-month follow-up (Lam et al., 2016). In a sample of patients with prostate cancer, among personality variables, optimism and self-efficacy were associated with lower emotional distress in disease’s early stage (Orom et al., 2015).

The ability to find meaning in cancer experience seems to be an important part of overall well-being for both patients and caregivers (Kim Y et al., 2011). However, other significant clinical factors as prognosis, radio-therapy, chemo-therapy, and illness stage account for survival. When controlling for them, optimism’s strength as a predictor of survival is invalidated (Coyne and Tennen, 2010; Schofield et al., 2016). In the other side, psychological adjustment to cancer is complex and optimism may contribute to adaptation and disease acceptance. As being optimist means having positive expectations about future outcomes, this may prompt positive affects and adjustment and may compensate loss-related affects resulting from unattainable goal disengagement (Lam et al., 2016). Further studies are needed to deepen multiple factors interaction in adjustment to cancer, focusing on its role acting in synergy with other psychological constructs.

#### Optimism and Cardiovascular Diseases

Optimism may influence cardiovascular health indirectly influencing health behaviors such as smoking, dietary habits, exercising, and adherence to treatment, both pharmacological and behavioral (Nabi et al., 2008; Tindle et al., 2010). The meta-analytic study of Chida and Steptoe confirmed that positive psychological well-being is associated with reduced cardiovascular mortality in healthy subjects and it is related to a better outcome in ill-being people. They considered positive affects (e.g., joy, happiness) as separated from positive dispositions (e.g., optimism, hope): authors hypothesized that the former may have a closer association with central nervous system modulating, for example, heart rate variability and the neuroendocrine system, while the latter seems to be more relevant in modulating coping strategies in stressful situations (Chida and Steptoe, 2008).

Studies on effects of optimism and positive psychological constructs on objective medical outcomes led to other consistent findings. Analyzing data from the Women’s Health Initiative, Tindle et al. (2009) found that optimism is associated with a reduced incidence of coronary heart disease (CHD) and total mortality. Moreover, comparing at baseline optimists to cynical hostile women (all free of cancer and cardiovascular disease), authors showed that the former had a better profile of protective factors for CHD, such as socioeconomic status and personal habits (e.g., no smokers and physically active women), and reported a better health condition (e.g., no diabetes mellitus, no hypertension, no high cholesterol, no depression). Furthermore, optimistic women were reported to have lower rates of total mortality, due to all causes (i.e., CHD-related, cancer-related, related to other cardiovascular diseases), rather than cynical hostile women (Chen et al., 2005; Tindle et al., 2009). Davidson et al. (2010) found a positive association between positive affect and risk of 10 year incident CHD (Davidson et al., 2010); similarly emotional vitality and optimism, as well as emotions self-regulation, have been found to be associated with reduced risk of incident CHD (Boehm et al., 2011; Kubzansky et al., 2011; Kim et al., 2014). Still, recent studies found, and confirmed, that optimism is a protective factor against stroke (Kim ES et al., 2011), reduces re-hospitalizations following coronary artery bypass graft surgery (CABG), even controlling for depressive symptoms (Scheier et al., 1999; Tindle et al., 2012).

Regarding indirect influences on health behaviors, optimism may promote subjective well-being and protective behaviors by fostering positive expectations about the outcome. In CABG patients baseline optimism, including time as a variable, predicted less pain (Mahler and Kulik, 2010). Similarly, in heart transplant patients optimism was significantly related to better mental post-transplant Health Related Quality of Life (Myaskovsky et al., 2006). Finally, in acute coronary syndrome (ACS) patients, optimism predicted low risk of depression, smoking cessation, and better dietary habits at 12-months after ACS (Ronaldson et al., 2015).

In conclusion, optimistic dispositions are associated with better cardiovascular health and reduced cardiovascular mortality and morbidity and with better subjective well-being, independently from socio-demographic status and psychological states like depression or anxiety (Tindle et al., 2010; Boehm and Kubzansky, 2012; DuBois et al., 2012, 2015).

#### Optimism and Respiratory Failure

Studies investigating the association between optimism and Chronic Obstructive Pulmonary Disease (COPD) are still scarce.

Popa-Velea and Purcarea (2014) investigated the role of optimism in determining perceived pulmonary function and HRQoL in COPD patients (*n* = 54). What emerged is that patients high in optimism and self-efficacy perceived less functional impairment than patients with low optimism and self-efficacy, with similar clinical biomedical parameters of pulmonary function. Being optimist, in addition to increasing subjective well-being perception, may stimulate health behaviors, such as following treatment programs or modifying life habits. Indeed, as for most chronic diseases, in COPD the motivation to engage in self-care is indispensable, which may be favored by several factors: self-efficacy, sense of agency, coping strategies, hope, knowledge, social support, and optimism (Kaplan et al., 1984; Scherer and Schmieder, 1997). As to our knowledge, one paper only deals with optimism and self-care in respiratory failure. Optimism and hope resulted associated with better self-care in COPD patients attaining a rehabilitation program (*n* = 68): being optimist fostered engagement with adaptive coping strategies and adherence to treatment (Alberto and Joyner, 2008).

#### Optimism and Multiple Chronic Conditions: Aging with Multimorbidity

Multiple comorbidity, a common factor in older adults, deeply affects health outcomes: predict mortality hospitalizations and costs, impact health-related quality of life, cause psychological distress, depression, and disability (Marengoni et al., 2011). High levels of dependence and/or institutionalization in the elderly are generally associated with a decreased quality of life. Kostkafound that dispositional optimism, as well as healthy locus of control (attributional optimism) and self-efficacy, are correlated with a higher level of quality of life independently from the environmental circumstances in which elders live (Kostka and Jachimowicz, 2010). Previous studies in aged population demonstrated that dispositional optimism is a predictor of less mortality and less permanent institutionalization controlling for age, gender and health measures (Pitkala et al., 2004), and that it is associated with healthier lifestyle and dietary habits in men aged 64–84 years (Giltay et al., 2007). In older patients with osteoarthritis, pessimism was associated with less social support and higher social strain, and indirectly influenced life satisfaction. Moreover, social support decrease and social strain were predicted by levels of pessimism after one year follow-up (Luger et al., 2009). Furthermore, high levels of optimism in old men predicted less loneliness feelings, despite aging-related events such as health threats, bereavement, isolation, loss of autonomy (Rius-Ottenheim et al., 2012).

Recently, dispositional optimism has been found to be related to better QoL and less emotional distress (i.e., anxiety and depression) in old patients (mean age 68.4 ± 10.2) with Parkinson’s disease (Gison et al., 2014).

The aging of the population is becoming a worldwide concern, where the main aim is not to increase life expectancy (i.e., years of life), but to improve the average healthy life years, that is to increase disability-free and morbidity-free years of life (EHLEIS, 2015). Optimism studies may provide useful information in this direction, helping to look inside the “black-box” contents, from psychological variables, to clinical variables and neuroendocrine, inflammatory, and immune responses (Ryff et al., 2004; Steptoe et al., 2005). As an example, in middle-aged and elderly Japanese people the presence of *ikigai*, intended as a positive psychological factor which comprehends the hedonic and the eudaimonic facets of well-being (e.g., joy of living, life worth living, benefit of being alive), has been demonstrated to be influential in reducing risk for all-causes mortality (Tanno et al., 2009).

## Conclusion

Literature on the protective role of optimism on health status is now noteworthy and is often characterized by sound methodology, good sample size and follow up at 6–12 months. As to optimism’s explicative role, different hypothesis have been formulated. It may stimulate adaptive coping strategies that foster seeking social support and consequently enhance subjective well-being, it might directly promote protective health behaviors and it might influence also immune responses and the neuroendocrine system modulation. Furthermore, an optimistic life orientation may sustain positive mood and protect mental health. However, the debate is still open upon optimism’s role in the mind–body–environment interrelationships.

Optimism is mainly described as a trait aspect of individuals, but it is not clear if optimistic aptitude toward life may change in lifetime. Developmental psychology research, longer longitudinal and health psychology studies specifically focusing on child, adolescent and informal caregivers should be added to the research agenda.

The mechanisms underlying the preventive effects of dispositional optimism still remain not completely clear and differences and commonalities between disease types are still unknown. Optimism is in its growing and significant scientific phase. Being aware not to back slide in an unsophisticated and exclusively hedonistic view, positive psychology research still holds unexplored fields of science which deserves a future glance.

## Conflict of Interest Statement

The authors declare that the research was conducted in the absence of any commercial or financial relationships that could be construed as a potential conflict of interest.
